# "The Hospital" Nursing Section

**Published:** 1906-05-05

**Authors:** 


### The Hospital.

### Nursing Section.

Contributions for "THE HOSPITAL," should be addressed to the EDITOR, "THE HOSPITAL" NURSING SECTION, 28 & 29 Southampton Street, Strand, London, W.C.

No. 1,023.—VOL. XL. SATURDAY, MAY 5, 1906.

### Notes on News from the Nursing World.

### HONOURS FOR NIGERIAN NURSES.

THE King has sanctioned the selection by the Chapter-General of the Order of St. John of Jerusalem in England, as honorary nursing sisters, of Mrs. Duncan Urquhart and Miss Margaret Manson Graham, two European nurses who have been in the employ of the Government of the Protectorate of Southern Nigeria since 1895. Their names were brought to the notice of the Chapter-General by Mr. Lyttelton, the principal medical officer of Southern Nigeria having called the attention of the late Colonial Secretary to the devotion and self-sacrifice shown by them on all occasions, and especially during the Aro expedition when they were engaged in the field, first at the base hospital at Ito, afterwards at Aro-Chuku, and finally at Bendé, where their services were of the greatest value. The decoration they are now entitled to wear consists of a Maltese cross of white enamel, embellished in silver on a black ground, the whole being mounted in silver, suspended from a black watered silk ribbon.

### OUTDOOR UNIFORM.

A CORRESPONDENT, who describes herself as "Fever Trained," writes from abroad to suggest that a society should be formed to supply badges to *bona-fide* nurses, the badges being only obtainable through hospitals. Our correspondent recognises that there are reasons against Registration, but thinks that "there is no hospital in the British Isles which would not join in a movement of the kind she proposes." We are afraid that she takes a very optimistic view of the situation, but her letter in which she indicates her ideas as to how her scheme might be carried out is noteworthy as showing the trend of opinion among trained nurses who do not at all agree with the view that to be seen in the streets in nursing costume is derogatory.

### A NOTABLE JUBILEE.

PREPARATIONS are being made to celebrate the Jubilee of the London Biblewomen and Nurses' Mission, which was founded in 1857 by the late Mrs. Ranyard, better known to the outside world as L. N. R., author of "The Book and Its Story." At the outset the workers consisted of Biblewomen, the Mission being affiliated to the British and Foreign Bible Society, but in 1868 district nurses were added to the Mission, in order that—to use the words of the founder—"hospital work might be undertaken outside all hospitals," one of the earliest subscribers to the fund being Miss Florence Nightingale. In its early days only a short hospital training was required, but this has been increased in recent years to a standard which ensures efficiency, and there are now 68 sisters and nurses employed in connection with the organisation. An appeal which is made on behalf of the Jubilee Fund is signed by several representative men, including Sir William Broadbent, Dr. Clifford Allbutt, Dr. Cullingworth, and Sir Thomas Smith, and at the first public meeting in commemoration of the Jubilee, which was held at the Æolian Hall, Bond Street, on Monday, June 18, Sir Frederick Treves will preside. The second public meeting is to take place in February next year, and the Bishop of London has consented to speak, if engagements permit.

### ON DUTY FOURTEEN HOURS.

A VERY discreditable state of affairs at Ashby-de-la-Zouch came to light at the last meeting of the Guardians, in consequence of the resignation of one of the nurses. The cause of her resignation, it was intimated, was that she was on duty from 6 A.M. to 10 P.M., and, if required, had to get up during the night. It was added, on her behalf, that she would not have thought of leaving if there had been a night nurse. Several of the Guardians, including the Chairman, admitted that the hours were very long, and one of them observed "that if there were a trade union among nurses it would not be allowed." But the Committee appointed to consider the question, cannot surely arrive at any other decision than that no nurse in their employ shall in future be permitted to remain on duty for fourteen hours, whether she is relieved of night work or not.

### ALLEGED OPENING FOR ENGLISH NURSES IN BELGIUM.

UNDER the title of "A New Field for Hospital Nurses," an article appears in a weekly contemporary in which it is urged that there is great scope for English nurses in Belgium. We have not the least wish to call in question the good faith of the writer, who has doubtless been impressed by difficulties experienced by individuals in securing the services of a trained nurse; but we take the earliest opportunity of warning our readers neither to rush across to Brussels on the off chance of securing an engagement, nor of risking their capital in the nursing institution which, it is affirmed, is badly needed in Belgium, unless or until they can obtain much more authentic information than is given in the columns of our contemporary. It would be possible by communicating with English medical men in Belgium to obtain reliable data.

### FOUR YEARS' TRAINING AT A POOR-LAW INFIRMARY.

The Birmingham Guardians have decided that probationers in their Infirmary shall, in future, be required to serve for four years, instead of three as hitherto, and that they shall be paid £20 for the fourth year. We shall not be surprised if the four-years' period of training is adopted in several other large Poor-law Infirmaries at an early date, especially in those which are recognised by the Central Midwives Board and midwifery training is included in the curriculum.

### A CATASTROPHE AT DERBYSHIRE INFIRMARY.

At Derby on Friday an inquest was held at the Derbyshire Royal Infirmary by the Coroner on an infant of nine weeks old. The evidence showed that the child was taken to the Infirmary on Thursday morning to undergo an operation, and that it occurred the same afternoon in the presence of three medical men and a sister. During the operation the doctor who gave the anæsthetic noticed that the child's breathing was bad, and he called to the sister to give the child two minims of strychnine. Standing on the table were two bottles exactly alike, one containing strychnine and the other morphia. The sister took up one, and, seeing that it contained strychnine, she prepared the syringe. The anæsthetist, noticing that the child was getting worse, called out, "Hurry up, sister"; and she, in her haste, snatched up the wrong bottle and administered morphia in mistake for strychnine. Almost immediately she recognised her mistake, and told the doctors. Antidotes were administered, and the child rallied for a time, but died in the evening. The sister, who was much affected in giving evidence, having stated that she had been six and a half years at Derbyshire Infirmary, and was previously at Grimsby Hospital, said that it was the first misfortune that had befallen her during her experience; and the medical testimony was to the effect that she was exceedingly careful, and that it was the custom to have every poison in a different kind of bottle. Subsequently, the secretary-superintendent stated that he "had examined every poison bottle in the institution, and found no second instance where a poison was in its wrong kind of bottle." The Coroner, in summing up, observed that "no blame could be attached to the nurse," and we are surprised that there should still be an institution which is at all lax in the matter of poison bottles.

### EXTRAORDINARY ACTION AT MEATH INFIRMARY

The Committee of Management of the Meath Infirmary have come to a decision which they venture to predict will be conducive to peace, but which is really subversive of authority. Both the matron of the Infirmary and the chief resident medical officer reported a staff nurse for neglect of duty and misconduct; but the Committee, having listened to a string of evidence, which, though contradictory, proved, by the admission of the nurse herself, that she had been guilty of an offensive practical joke on a patient, refused to act upon the statements of their responsible officials, and took upon themselves the task of trying to settle the question by directing the nurse to assume charge of another ward. This flagrant interference with the duties of the matron cannot in any circumstances be justified, and we are surprised that the Committee of the Meath Infirmary should have exposed themselves to the kind of criticism which is incurred by the Guardians of some of the inferior Irish Poor-law institutions.

### THE SALARY QUESTION AT EDMONTON.

At the usual meeting of the Edmonton Guardians a discussion took place on a recommendation of the Workhouse Committee that application should be made to the Local Government Board for their sanction to the increase of the nursing staff by the addition of not less than four assistant nurses at salaries of £24, rising £2 annually to £28. Some of the Guardians thought that £24 was too low a salary when a nurse has received three years' training, and a rise to £35 was also suggested, but in the result the recommendation was adopted, and it was decided, subject to the approval of the Local Government Board, to fill up two vacancies by the appointment of two of the nurses trained at Edmonton. The salaries which have been decided upon are the same as those offered by the Metropolitan Asylums Board to their first assistant nurses, after the latter have either received training for one year in a general hospital, or been nursing in a fever hospital for two years.

### DISTRICT AND PRIVATE NURSING.

Lord Yarborough, presiding at the annual meeting of the Grimsby and District Nursing Institution, referred to the change which had taken place in its constitution, and expressed his belief that, now it had become purely a charitable institution depending upon charity and public subscriptions, it would receive adequate support. The wisdom of the change cannot, at all events, be doubted. Grimsby people who are in a position to pay the fees of private nurses are amply provided for by the establishment of a new organisation in the old home of the Institution; and, with Miss Lunn as superintendent, the Queen's nurses at Grimsby are sure to find their work appreciated. Lord Yarborough specially eulogised the efforts of the lady collectors, who last year obtained over £200 in small subscriptions, this being a record sum. We are satisfied that these ladies will find it much easier to secure financial assistance now it is no longer possible to urge that the Institution can fall back on revenue from its private nursing branch to meet its expenses.

### THE PENSION FUND AND KENTISH NURSES.

On Thursday, last week, a well-attended meeting was held at the Kent and Canterbury Hospital, to listen to Mr. Louis Dick, Secretary of the Royal National Pension Fund for Nurses, who gave a lucid explanation of the advantages and reasons for joining. The Dean of Canterbury, Chairman of the Hospital Committee, presided, and amongst others present were the Chairman of the General Committee of the Nursing Institute, the senior surgeon to the Kent and Canterbury Hospital; Miss Messum, matron of the Kent and Canterbury Hospital; Miss McMaster, matron of

Chartham Asylum; Miss Alcock, matron of Feversham Cottage Hospital; Miss Dale, matron of Kennaway's Hospital Feversham; Miss Thompson, matron of the Borough Asylum; Miss Abbott and Miss May, Whitstable; Miss Bye, Sandgate; Miss Gurgn, lady superintendent of the Nurses' Institute, Canterbury, and a large number of nurses from Canterbury and the neighbourhood. The Dean heartily recommended the objects of the meeting, and, in conclusion, begged his hearers not to forget the wise old adage: "If youth could know what age will crave, then youth would every sixpence save." Miss Messum subsequently provided tea in the board-room. The Nurses' Institute has long been affiliated to the Pension Fund and many of the nurses are members.

### IMPERIAL MILITARY NURSING SERVICE.

We are officially informed that Miss S. G. M. Rogers has been appointed staff nurse in Queen Alexandra's Imperial Military Nursing Service. Miss S. G. M. Rogers has been posted to the Royal Herbert Hospital, Woolwich, on her appointment as staff nurse. The appointments of Miss E. Close and Miss L. M. Draper as staff nurses have been confirmed.

### GUY'S HOSPITAL MUSICAL SOCIETY.

The second, and final, concert of the season was given by the Guy's Hospital Musical Society on Tuesday evening. Since the last concert, in December, an amalgamation has been completed of the Nurses' Choral Society, Students' Glee Club, and the newly formed Orchestral Society. Appropriately enough, those responsible for the amalgamation determined to celebrate the event by giving the net proceeds of the concert to the Million Shilling Fund in aid of the hospital. The opening pianoforte duet, by Nurses Taylor and Clifford, was played with an artistic sense that deserved the hearty applause which it evoked. This was followed by two songs by W. S. Kidd, After a song by Sister Mary, the Choral Society gave two part-songs, "Ave Maria," and "Lullaby of Life." Amongst other items in the first part was a tenor solo accompanied by a humming chorus, which was much appreciated. Sister Lydia's song, "An Irish Lullaby," was received with prolonged applause, and the only encore allowed during the evening was given after a clever recitation by Sister Cornelius. The second part consisted of two items only: G. A. Ticehurst gave Grieg's "Holberg Suite." The *pièce de résistance* was reserved for the end in the form of Elgar's cantata "The Banner of St. George," and the Society is to be congratulated on the excellence of its performance. The solo was well taken by Nurse St. John, and the arduous task of accompanying was performed throughout by Nurse Taylor. The number of tickets sold for the concert has, we hear, established a record, so the Million Shilling Fund should shortly receive a nice little addition.

### ART AND DISTRICT NURSING.

An exceptionally interesting effort will be made on Wednesday, May 16, to aid the funds of the East London Nursing Society. By the kindness of Lady Portsmouth an exhibition and sale of watercolour drawings will be held at 16 Mansfield Street, Cavendish Square. It will be open from 11.30 A.M. to 6.30 P.M., and tea and coffee will be served to the visitors. We trust that this admirable expedient for raising the wind on behalf of a most deserving institution which is greatly in need of support, will be as successful as the promotors could desire.

### MEMORIAL HOME IN GLASGOW.

The "Alice Mary Corbett" Memorial Nurses' Home, which has been given to the Glasgow Samaritan Hospital for Women by Mrs. Polson, of West Mount, Paisley, has been handed over to the institution. It has been erected at a cost of £10,000, and was furnished at the expense of the donor. The Home contains three floors, and accommodation is provided for nearly thirty nurses, each one having a separate room. Convenient quarters are assigned to the matron.

### AN EXCELLENT EXAMPLE AT LOWESTOFT.

An unusual item in the receipts of the Lowestoft District Nursing Association, which has just held its annual meeting, is the amount received from visitors to Somerleyton Gardens. By the kindness of Sir Savile and Lady Crossley, their beautiful grounds are thrown open to the public, who acknowledge the courtesy by putting money into the boxes kept for the purpose. This admirable example might be widely followed at a period of the year when the charms of private gardens are so often enjoyed by outsiders. Many persons who take advantage of the privilege would gladly avail themselves of such an opportunity of acknowledging it if they had the chance provided for them. Lowestoft is happy altogether in respect to the management of its Nursing Association, for the staff are housed without cost by generous supporters of the charity, and in finance the balance is so far on the right side.

### SHORT ITEMS.

During the winter addresses have been delivered weekly to mothers and daughters at the Wyche Meeting House, Malvern, by Nurse Evelyn Hobday, on first aid in cases of sickness and accident at home, hygiene, and cookery for invalids, and last week at the conclusion of the series Miss Hobday was presented with a crocodile satchel in appreciation of her endeavours.—At Addingham, a small village between Skipton and Leeds, a social gathering including music and other entertainments has just been held in aid of the District Nurses' Fund, the substantial sum of £10 1s. being realised.—In the report to the Guardians of Kingston-upon-Thames upon the examination of some of the nurses at the Poor-law Infirmary, the examiner states that Miss M. R. Parsons obtained 100 per cent., and two other nurses 95 and 90 per cent. respectively, a result reflecting the utmost credit upon the authorities of the training school.—The Duchess of Buccleuch will preside at the annual meeting of the Westminster Hospital Ladies' Association, which takes place on Monday, May 21, at the Westminster Hospital, at 3.30 P.M., and the Marchioness of Salisbury at the annual general meeting of the University College Hospital Ladies' Association, to be held on May 23 at 4.30 P.M.

### The Nursing Outlook.

"From magnanimity, all fears above;
From nobler recompense, above applause,
Which owes to man's short outlook all its charm."

### RURAL WORKHOUSE NURSING.

The nursing in the sick wards of rural workhouses is often far to seek. Dr. Buckell, medical officer of the Romsey Guardians, at the last meeting gave the following account of what happens to patients in the Romsey Workhouse sick wards during the night. These wards, it would appear, are occupied largely by helpless patients, but there is no night nurse, with the following results: "The old people tumble out of bed at night and cannot get back by themselves; there is only one inmate on the women's side who is in the least capable of getting out of bed and helping, and she has complained to me that she hardly ever gets a night's rest owing to the trouble caused by the other old people. Many of the patients are 'wet' cases, and should be changed during the night if bed sores are to be avoided. Several times a fatal accident has only been avoided by what may be termed good luck." Every nurse and all who have to do with sick people will grasp the horror and misery entailed on the inmates by grave evils like those here described. Is it wonderful in such circumstances that the respectable poor have such a dread of the workhouse always?

The nursing staff is too small to enable both day and night nursing to be undertaken, hence the trouble described by Dr. Buckell. We have no doubt that the Romsey Guardians number some, and possibly many, excellent people, who desire to do their duty to the sick poor committed to their care. It is probable, however, that none of them have visited the sick wards at night, or that they have the slightest appreciation of the suffering and misery entailed upon the helpless inmates by the absence of proper nursing arrangements. Who, however, can read Dr. Buckell's report without experiencing a feeling of amazement and indignation that avoidable miseries of the kind described should be suffered by the helpless inmates of sick wards in rural workhouses under the present Poor-law administration? The Poor-law inspectors do their best to ameliorate a condition of affairs like that at Romsey wherever they find it, but in practice it will be seen that their influence for good cannot be great, or it would be an impossibility to find the condition of neglect, of which the Romsey case is typical, in any rural or other workhouse in this country to-day.

Mr. Baldwin Fleming, the Local Government Board Inspector, in his report on Romsey states that there are two infirmaries at some distance apart, and only two nurses—*i.e.* practically one for each infirmary. Thus there is no provision for adequate night nursing, although the nurses have a wardmaid to help them, and some further assistance has been rendered as regards the housework. Mr. Baldwin Fleming enforces the view that it is impossible for good nurses in the conditions described, without an undue strain upon themselves, to attend to the patients by day and night. In the result the Guardians decided to appoint one probationer and one nurse-attendant, in lieu of the assistant nurse who had resigned, and in this way it is hoped that some attendance may be given to the patients during the night.

We have reason to think that the Romsey case is by no means an exceptional one, and that the condition of affairs in many of the sick wards of rural and other workhouses is as bad as, if not worse, than that just described. It is certainly wrong for any public institution to retain sick inmates under the conditions reported by the inspector of the Local Government Board, and Mr John Burns might do good service to the poor if he were to give instructions to each of the inspectors to specially investigate the night conditions which prevail in sick wards throughout the whole of the workhouses in this country. We can quite understand that those of our readers who are accustomed to the excellent arrangements to be found in the majority of hospitals, and in the best Poor-law infirmaries, will find it difficult to believe that anywhere in England at the present time old and helpless sick patients in an enfeebled and paralysed condition are left entirely alone throughout the night. But Dr. Buckell's report proves that this condition has prevailed at Romsey for years! Fancy the misery and risk of tumbling out of bed and remaining on the floor, unattended and helpless, until the morning!

Seeing that these conditions are admitted to prevail, it is essential, on the grounds of humanity alone, that an immediate and searching investigation should be made into the nursing arrangements for sick wards in workhouses during the night and generally. It is further of great importance to the well-being of the ratepayers, and especially of their children suffering from infectious disease, that an inquiry should be instituted into the nursing arrangements of each of the multifarious so-called hospitals for infectious diseases which have been set up by health authorities in rural districts especially. A Departmental Committee, presided over by the medical officer of the Local Government Board, could not be better employed than in ascertaining the actual facts relating to the nursing of sick wards and local infectious diseases hospitals. Is it too much to hope that Mr. John Burns will appoint such a committee with as little delay as possible?

### The Care and Nursing of the Insane.

By Percy J. Baily, M.B., C.M.Edin., Medical Superintendent of Hanwell Asylum.

### I.—ANATOMY AND PHYSIOLOGY.

(*Continued from page 49.*)

*The Pulse.*—The different chambers of the heart on the right and the left side contract together—that is to say, the two auricles contract at the same time, and immediately after these, the two ventricles; there is then a slight pause, during which the auricles are being again refilled by the blood which is pouring into them from the veins. At each beat of the heart the same amount of blood is discharged from the left ventricle as from the right.

It will now be easily understood that the blood in the arteries is periodically augmented by the sudden discharge into them of the contents of the ventricles, and that thereafter no blood is received into them until the occurrence of the next ventricular contraction. Hence the blood in the arteries flows in waves, each wave distending the elastic walls of the arteries as it passes along.

It is this wave which constitutes the pulse, and the rapidity of the pulse therefore indicates the rapidity of the heart-beat. This varies at different ages. In young children the pulse beats at the rate of about 140 times a minute, and gradually diminishes until adult age is reached, when it averages 72 per minute in a man and about 80 in a woman. In old age it again becomes more rapid. The rate at which the heart beats, and therefore also, of course, the rate of the pulse is increased by muscular exertion, mental excitement such as is caused by sudden and unexpected pleasure or sorrow, fright, etc., etc., the taking of food—various drugs, especially alcohol—and increased temperature (fever); on the other hand violent mental emotions will sometimes cause the heart to stop beating. This is the cause of death when one is said to die of fright or sudden grief.

It is necessary to explain here that the blood flows much more rapidly in the larger arteries than in the smaller arteries and capillaries. The reason of this is that, although, as the arteries divide up, each resulting branch is individually smaller than the vessel from which it arose, yet the number of them is so enormously increased that the total capacity of the minute arteries and capillaries is much greater than that of the larger arteries. One may compare the blood course from the aorta to the capillaries to that of a river which gradually widens out towards its mouth. In the narrow upper reaches of the river the stream is rapid, and as it widens out towards its mouth it flows along more and more slowly. So greatly is the blood channel increased in area in the capillaries that the pulse wave is here altogether lost, and the blood flows through them in one continuous steady stream. As the capillaries unite and form the minute veins, and these again the larger ones, the rapidity of the blood current necessarily again increases as the total blood channel becomes gradually narrowed down; but the current in the larger veins is never as rapid as that in the larger arteries, as the capacity of these veins is greater than that of the larger arteries. Again, since the pulse wave is lost in the capillaries, there is, of course, no pulse in the veins, the flow of blood in these vessels being continuous and not jerky.

The blood of any individual may be regarded as being always the same in total amount. The quantity, however, which circulates through any particular organ varies very greatly in amount from time to time, depending upon the requirements of the organ. When we are using our muscles, as in walking or cycling, the quantity of blood flowing through them is much greater than when they are at rest; so when we are thinking our brains receive more blood than is the case when we have nothing to think about; and after taking a meal, while in other words we are digesting our food, the blood supply to the various parts of the alimentary canal is increased. The amount of blood which is supplied to any of our organs is under the control of the nervous system, and the regulation of the supply is brought about by the involuntary muscular fibres which, as we have already seen form, in part, the walls of the blood-vessels, and especially of the smaller arteries. When an organ requires much blood, these involuntary muscular fibres relax, and consequently the small arteries become wider, and so allow a greater amount of blood to pass through them. When the amount of blood required becomes less these fibres contract, and so diminish the calibre of the small arteries, and thus lessen the amount of blood passing through them. The act of blushing depends upon the same mechanism. We all know that in certain emotional states the amount of blood in the skin of the cheeks varies. If the state is one of shame the cheeks become red (blush), while those who give way to passionate rage often become blanched and pale—white with rage as we say. In blushing an impulse travels from the brain through the nerves which supply the involuntary muscular fibres contained in the small arteries of the cheeks, with the result that these relax, while during a fit of passionate rage the impulse which travels to these fibres causes them to contract so that the amount of blood passing through them is greatly diminished. It will be more easy to understand the nervous control of the blood-vessels when we come to speak of the nervous system.

### 6. The Digestive System.

During the whole period of life our tissues are always doing work. The work which a tissue does (energy) is, of course, its own special function. For example, the muscles produce motion, the brain thought, the various glands in the body their own particular secretions, etc., etc. But the performance of work entails waste of substance, and depends upon oxydation or burning. Now, whenever anything is burned—wood or coal, for instance—certain events follow with which we are all familiar. Heat is produced, while the coal or wood gradually disappears, passing away as smoke or ignited gases

(flames). After the process is complete some ashes remain behind, which differ altogether in appearance, etc., from the original coal. A similar process may be said to occur in our tissues; the substances of which they are composed are continually undergoing oxydation, or in other words, are being slowly burnt; and as a result of this heat and various waste matters (ashes) are produced, while the materials of which the tissues are composed are used up. The greater the amount of work done the more rapid is the burning, and so the greater is the amount of heat and ashes produced; and therefore, of course, the greater also is the wasting or wearing out of the material. Our tissues are therefore continually losing substance, and hence as long as life lasts they require to be replenished and fed. We all know that if we cease to eat we become thin and waste away, and ultimately die of starvation.

We have already seen that the food is carried to the tissues from the alimentary canal by the blood. Before the food can be absorbed into the blood it must undergo many changes so as to render it soluble and absorbable. These changes are accomplished by the process of digestion. As we shall see immediately, the secretions of innumerable glands are poured into the alimentary canal. Some of these glands lie outside the digestive tube and their secretions are conveyed into it by means of ducts or channels, for instance, the salivary glands and the liver and pancreas; others are minute structures which lie embedded within the mucous membrane which lines the interior of the tube throughout its whole extent. The food as it passes along the alimentary canal is brought into contact with and is acted upon by these various secretions or digestive juices which dissolve the solid parts and reduce them to a condition which enables them to be readily absorbed into the blood.

By the process of digestion then, the various foods which we take are broken down and dissolved so that the particles (molecules) of food which are actually absorbed into the blood differ widely in many respects from those which we actually eat. If we take a piece of muscle (ordinary lean meat—mutton or beef) as an example, we find that this is crushed and torn to pieces by the teeth (mastication) and is, after being swallowed, reduced to a soluble state by the pepsin in the stomach. No one would now recognise, by the ordinary methods of observation, that this solution was muscle. Yet all the constituents of muscle are there, and when these are carried to the muscles of our bodies by the blood, they are again built up into actual muscle tissue. This latter process whereby the food particles or molecules as they exist in the blood are again converted into the material of the tissues, muscle, bone, brain, etc., etc., is known as the process of assimilation, and is in every respect as important a process as that of digestion. It is, then, by the process of assimilation that the worn-out tissues are renewed. It is, perhaps, not easy to understand that we, like our clothes, are always wearing out. Our tissues do not assume a shabby appearance, nor do they wear into holes, because as fast as they wear out the worn parts are repaired by assimilation. The processes of destruction and repair are being carried out at one and the same time. While we are young and growing, assimilation is in excess of the destruction or disintegration, and hence the total bulk of the body increases. After we are fully grown the two processes are equal in amount and we maintain a more or less fixed bulk or weight. As we grow old, or when attacked by various diseases, the destruction is in excess of assimilation and we lose weight and become thin. This process of assimilation is dependent for its proper performance, among other things, upon healthy nervous impulses received by the tissues through the nerves from the brain; and hence it is that we frequently find that patients suffering from brain disease, especially general paralysis, waste away to mere skeletons although their appetite may be almost voracious.

### The Nurses' Clinic.

### PERINEORRHAPHY CASES.

Perineorrhaphy is a plastic operation which is done to repair an old tear in the perineum.

Rupture of the perineum may occur during childbirth. It is most likely to take place during a first labour, or instrumental labour. In some cases the medical man in attendance may succeed in bringing the parts together at the time of rupture, with a few sutures; but, failing this, the raw edges become healed, as time goes on, without uniting, and if the tear be extensive the patient suffers a good deal of discomfort which calls for operation.

The complications which usually arise from ruptured perineum are prolapse of the uteres and perhaps of the bladder or rectum along with part of the vaginal wall.

The patient may lose control of the bladder or suffer from incontinence of the fæces.

The preparation of the patient for operation will be as follows:—

An aperient must be given about midday the day before operation, and an enema (soap and water) during the evening, so as to secure free action of the bowels. The parts must be shaved and thoroughly cleansed with soap and water. A bath, and a vaginal douche of some antiseptic solution will be necessary.

On the morning of the operation the patient will require to have another soap-and-water enema early. A wash-out enema and vaginal douche should be given within an hour before operation.

Breakfast should be light, tea and toast, and ought to be given at least four hours previous to operation. The patient must pass urine immediately before going to the operating theatre.

The principal instruments, etc., required will be scalpel, scissors (angular, straight, and curved on the flat), perineum needle with long handle, curved needles and half-curved needles, with needle-holding forceps, twelve pairs of pressure forceps, two pairs of dissecting forceps, catheter, douche nozzle and irrigator filled with antiseptic solution, swabs of sterilised gauze, pad of sterilised gamgee and T bandage, sterilised towels, ligatures of artery silk, and strong silk worm-gut sutures. The patient should wear a

**THE NURSES' CLINIC**—*Continued.*

warm dressing-gown and stockings, and a blanket should be spread over her chest. She will be operated on in the lithotomy position, the legs being supported on each side by crutches.

When the operation is over and the patient is put to bed, the legs must be fastened firmly together with a bandage so as to keep the wound at rest, a pad of wool being placed between the knees to save friction, and a pillow underneath so as to keep the knees in position.

There may be slight hæmorrhage for the first few hours after operation, in which case the dressings may require frequent changing.

Vaginal douching will be necessary night and morning. Patients after this operation may be unable to pass urine themselves for several days, in that case the catheter must be passed, but not oftener than eight-hourly, great care and surgical cleanliness being observed by the nurse, the wound being washed up each time with an antiseptic, and fresh dressing applied.

An aperient is generally given about the second day after operation.

Each time the bowels move or urine is passed the wound must be gently syringed with some weak antiseptic solution (boracic or perchloride), dried with sterilised swabs, and fresh dressing applied.

The surgeon usually removes the sutures about the end of a fortnight.

The patient will probably be allowed to get up in about three weeks' time from the operation.

Light ordinary diet may be given almost from the first after the chloroform sickness has passed.

The principal thing to be observed in the nursing of all operations of this class is the keeping of the parts thoroughly clean.

### Incident in the Life of an Irish Nurse.

### MY FIRST DAY IN HOSPITAL.

THE first of November, 18— was a red-letter day in my life, for it was then I embarked upon my nursing career, and I am still at work in the field.

I arrived at the Nurses' Home in the afternoon, and was handed over by the Matron to a demure little nurse, who was told to help me to get into my uniform, and take me over to the hospital when ready. She was kindness itself, although she did brush my hair smoothly back off my forehead and made my cap unbecomingly flat and broad like her own. She remarked that no fringes were allowed, and she feared that my dress was too long and my apron too short; which I thought were trifling matters. However, I soon learnt that a uniform dress trailing the ground and an apron reaching a little below the knees were matters of grave importance. These obstacles, however, did not hinder my having a heart full of confidence and hope as I cheerfully tripped across the cold, wet garden with nurse as chaperon to the hospital. I did not question then what the life before me meant, nor what it was going to bring. I wanted work, and meant to do it with my might. We soon arrived at the door of the Lady Superintendent's room. A single knock on the brass knocker brought a "Come in," and I found myself standing before my chief. She shook hands with me, hoped I should make a "good nurse," told me to have a tuck put in my skirt and my apron lengthened before the next day, and also added that I might get a plain silver brooch for my collar, instead of the gold one I was wearing. She then looked at my feet, and was shocked to find that I was wearing fancy shoes with buckles and high heels, for she said I was to get "sensible shoes, with broad toes and flat, noiseless heels." I left her presence not quite so self-satisfied as I had been a few minutes previously, for I had begun to realise the importance of the fitness of a nurse's uniform.

My next experience was in the children's ward, where I was introduced to the sister as the "new pro." She gave me a genuine North of Ireland welcome, and took me round each of the cots, telling me the name and history of each little patient. One bright boy, "Charlie," I shall never forget. He had hip disease, and been in hospital for two years. He got quite confidential after a few minutes' talk, and told me to ask him anything I wanted to know. "You see," he said, "I have been here so long I know everything. Sister says I am as good as any of the nurses."

In a short time I was very glad of the little boy's experience, for the accident bell rang, and I went with sister to the theatre. Boracic powder was wanted, and she sent me to the ward for it. I kept repeating the difficult word all the way, lest I should forget it before I could get back to Charlie's bed. Amidst peals of laughter from the children I called out to him, "Borakik, Borakik," where shall I get "Borakik"? The little fellow raised himself on his elbow and pointed to a large flour dredger which stood on the table. I seized it and ran (I had not yet learned the lesson that a nurse should never run when on duty) to the theatre in doubtful uncertainty, for I thought the child must surely be making a fool of "the new pro." It is hardly needful to add that I was always called "Nurse Borakik" in the children's ward after that.

In a short time another accident arrived—a child in convulsions. A bath was ordered immediately, and I was sent to the kitchen with all speed for hot water. As I always understood the kitchen to be a downstairs apartment, I dashed, can in hand, for the broad stone staircase. Down, down, down I went, thinking that the steps would never come to an end. At last I found myself in a huge kitchen. A cross-looking cook met me at the door and asked me what I wanted there. She said, "Don't you know very well you're breaking the rules; you'll be killed if you're caught here." I explained to her that I wanted hot water for a child in convulsions, and must have some, but she told me to go back to my own kitchen; "divil a drop of hot water" I'd get from her. So I retraced my steps as fast as I could, feeling hopelessly puzzled as to what I could do next, when fortunately I met a nurse on the stairs who directed me to the ward kitchen, where I filled my can in breathless haste and ran back to the ward to find the house surgeon and sister waiting anxiously, if not too patiently, for my arrival. Alas for too much haste! Alas for high-heeled shoes, and alas for the poor little patient without his bath all this time! Down I went a "cropper" on the polished floor, hot-water can and its contents on the top of me. "Poor little nurse, are you hurt?" I heard someone say; but I did not wait to answer. I was soon on my feet again, and, amidst the laughter of the children, ran out of the ward to the kitchen, replenished the water can, and was back in a trice.

That night, as I sat on my bed shortening my frock and lengthening my apron, I stitched many a tear into my work, for I had heard it whispered, "She will never make a nurse," and I feared it must be true. But, thank God, time has proved that I was not a failure, and I have even risen to the dignity of a matron.

### Queen Victoria's Jubilee Institute for Nurses.

Her Majesty Queen Alexandra has been graciously pleased to approve the appointment of the following to be "Queen's Nurses" to date April 1, 1906 :—

England and Wales. — Elizabeth Stinson, district training at Bermondsey; Ada Bracelin and Emma Maude Norrie, Birmingham (Newhall Street); Dora Margaret Laycock, Blackburn; Ann Susan Barnett, Ellen Elizabeth Pattenden, Ellen Ross, Lucy Seward, and Rachel Augusta Williams, Bloomsbury; Emily Hazlitt, Bolton; Beatrice Low Booth, Ethel Mary Clay, Winifred Annie Orpwood, and Lily Harriet Workman, Brighton; Harriet Clarke, Camberwell; Hannah Clara Dando; Kate Eleanor Hampton and Robina Wilson, Cardiff; Amy Kate Baughurst and Ethel Mellor, Chelsea; Mary Walford, E. London (Central Division); Ellen Cattell, and Sarah Sweatman, E. London (Southern Division); Olive Goddard and Mercy Watkins, Gloucester; Christine Raine, Hammersmith; Eva Stevens, Hants Nursing Association; Emily Isobel Branscombe, Amy Carlton, and Lois Blanche Watkins, Kensington; Violet Jane Webster, Leeds (Central Home); Minnie Elizabeth Butcher, Lydia Sabra Dunbar, and Lily Eiddon-Jones, Liverpool (Central Home); Elizabeth Gordon Irvine, Liverpool (Derby Lane Home); Katherine Jane Jones and Emma Wherritt, Liverpool (Kirkdale Road); Sarah Kneale, Liverpool (Overton Street); Annie Meeson, Liverpool (Shaw Street); Ada Arkwright, Manchester (Bradford Home); Blanche Sykes; Harriett Ellen Browne, Rosa Beatrice Keeble, Ada Elizabeth Marsdin, and Florence Maude Pearce, Norwich; Margaret Ellen Marsden, Oxford; May Holden, Paddington; Alice Louisa Hooker, Portsmouth; Eliza Harriet Crosier, Salford; Mabel Dadd, Alice Glanville, Eliza Constance Annette Middleton, Isobel Francis Sailly, and Florence Elizabeth Slack, Shoreditch; Gladys Mary Alwn Lloyd, Southampton; Millicent Neilson, Harriet Pulford, and Janet Wilcock, Stockton; Minnie Augusta Stout, Sunderland; Clara Julia Lambert and May Nicholson, Walworth.

Ireland.—Mary Ellen Leonard, St. Lawrance's Home, Dublin; Clara Armstrong, St. Patrick's Home, Dublin.

Scotland.—Elizabeth Brown, Agnes Wightman Graham, Henrietta Elizabeth Lowe, Harriett Magowan, Sara Martin, Agnes Weir, and Alice Jane Harley Williamson, Edinburgh.

### Central Midwives Board.

A Meeting of the Board was held on Thursday last, and there were present Dr. Champneys (in the chair), Mr. Ward Cousins, Dr. Dakin, Mrs. Latter, Miss R. Paget, Miss Wilson, and Mr. Parker Young.

The Secretary reported that at the April examination (which was for London only) 215 candidates sat. Of these the percentage of failures was 21.4. Sixty-five of the candidates had previously failed and forty-five of these candidates passed.

The report of the Standing Committee was then taken. With reference to a letter from Dr. Walford, M.O.H., Cardiff, forwarding copy of a resolution passed by the Local Supervising Authority of Cardiff, asking the Board to make Cardiff one of the provincial centres for the Board's examinations, it was decided that it is not desirable to constitute Cardiff an examination centre at present. That Dr. Walford be asked to inform the Board whether, in the opinion of the Local Supervising Authority for Cardiff, candidates not understanding English could write papers in Welsh; and whether there are facilities for teaching such candidates in Welsh, and, if so, where? That Dr. Walford be asked to furnish a list of examiners competent to examine in Welsh. That Dr. Walford be informed that the above resolutions are not to be taken as in any way prejudicing the ultimate decision of the Board.

In view of a letter from Dr. Toogood, Medical Superintendent of the Lewisham Infirmary, stating that the improvements required by the Board had been carried out, it was resolved to recognise that Infirmary as a training school.

A letter was read from Ruth Drakeford, certified midwife, complaining of the increased fees charged by doctors when called in to a case attended by a midwife. The letter stated that doctors, who objected to the work of certified midwives, were in the habit of warning patients beforehand that if called in in an emergency by such midwife, their fee would be double what they would otherwise charge. This practice was a great deterrent to the employment of trained midwives.

Mr. Ward Cousins spoke strongly on the evils attendant on this question of payment to doctors if called in by midwives in accordance with the Act, and said he had known cases where the midwife herself had been obliged to pay the doctor's fee. He thought that the Board ought to express some opinion on the point. It was pointed out that the Board had no power in the matter, nor had the county local supervising authorities. Liverpool and Cardiff had made arrangements for the payment of doctors, but, being Borough Councils, they had a freer hand than the County Councils, as their accounts were not subject to the Local Government Board auditor.

After some discussion it was decided that the best way of dealing with the matter was to refer to the Standing Committee the drawing up of suggested amendments to the Act. Amendments had been proposed to the late Government, but the Board felt that it might be now opportune to approach the present Government. This question of doctor's fees should have a place in the amendments.

A letter from Mr. C. J. Wright, one of the Board's examiners, as to additional fees for examiners in certain cases was considered. The letter pointed out that when long journeys had to be undertaken no return was made to the examiner for the expenditure of his time. It was decided that Mr. Wright be informed that the Board is not prepared to disturb the existing arrangements so soon after inauguration; and that the Secretary be instructed to calculate the average duration of the journeys undertaken by the provincial examiners at each examination.

Shoreditch Union Infirmary was approved as a training school.

It was decided to form a sub-committee to consider the question of new offices for the Board. The Chairman, Miss Wilson, and Mr. Parker Young were elected members of the Committee, and a special meeting of the Board to receive their report was fixed to take place after the Penal Cases Committee on May 3. The next monthly meeting of the Board was arranged for May 24th.

### To Nurses.

We invite contributions from any of our readers, and shall be glad to pay for "Notes on News from the Nursing World," "Incidents in a Nurse's Life," or for articles describing nursing experiences at home or abroad dealing with any nursing question from an original point of view, according to length. The minimum payment is 5s. Contributions on topical subjects are specially welcome. Notices of appointments, letters, entertainments, presentations, and deaths are not paid for, but we are always glad to receive them. All rejected manuscripts are returned in due course, and all payments for manuscripts used are made as early as possible after the beginning of each quarter.

### Everybody's Opinion.

[Correspondence on all subjects is invited, but we cannot in any way be responsible for the opinions expressed by our correspondents. No communication can be entertained if the name and address of the correspondent are not given as a guarantee of good faith, but not necessarily for publication. All correspondents should write on one side of the paper only.]

### POOR-LAW NURSES AND SUPERANNUATION.

"Ignoramus" writes: In your current issue I read with interest your Note on Poor-law nurses and superannuation. You state that the Local Government Board have modified their decision, and that a nurse who has contracted out, may, on taking up higher office, again come under the Act. I myself in 1898 as a probationer contracted out, and I have regretted doing so since. After my training I was promoted to staff nurse, subsequently ward sister, and at present I hold office as home sister. May I now claim to come under the Act? [Yes.—Editor, The Hospital.]

### NURSES AND TEMPERANCE.

"M. H." writes: May I bring under the notice of your readers a meeting of the Women's Union of the Church of England Temperance Society, to be held in Caxton Hall, Westminster, on Thursday, May 10, at 3 p.m. Mrs. Temple will preside and the speakers will be Sir Victor Horsley, on the use of alcohol and drugs in physical and mental strain; and the Rev. E. F. Russell, Chaplain to the Guild of St. Barnabas. Questions and discussions will follow the addresses. Such a subject, dealt with by such speakers, must be of the greatest interest to nurses, and I venture to hope that you can spare room for this letter, so that the meting may be more widely known in the profession. Tickets may be had free by applying to the Secretary, Church of England Temperance Society, The Sanctuary, Westminster, S.W.

### A NURSE ASKED TO BE NURSEMAID.

"Justice" writes: Will you kindly give me space in your paper to ask some of my fellow-nurses what their opinion is, and if they ever had a like experience. A certificated nurse attached to a private nursing institution in London was asked to go to a case, a baby who was not ill, to look after it and take it out for airing in a perambulator until the lady could decide upon a nursemaid. The nurse refused, as she has a strong objection to taking a nursemaid's duties. Unfortunately the matron was away at the time, but when she returned the nurse was called upon to resign for "rudeness and insubordination." There were other nurses in the home at the time, but they were not asked to undertake the work, and so escaped being called upon to resign, though I am almost sure that they would have refused to comply with the request.

### CHANGES AT BARROW.

"A Barrow Guardian" writes: My attention has been drawn to a note in your issue of the 14th, which is far from true, and may unjustly prejudice or limit our selection of officers in future, and should in all fairness be corrected. Two officers tendered their resignation at the meeting referred to, a nurse and assistant matron. This called for a few remarks from one of the Guardians, very incorrectly quoted in every particular. He was quickly calmed by the assurances of the Chairman, whom you quote, and correctly so. Going back three years as stated, 15 officers have left the service of the Board, many of whom might not have had the chance had we been half as bad as your note infers. Of these nine were nurses and six on the workhouse side. Five nurses left closely together owing to a change of policy on the part of the Board improving their status by engaging skilled nurses only. Two left to go abroad, and two to better positions. On the workhouse side two to better positions, two to be married, one to go into business, and one for health reasons. The only correct part of your note is the last, where you say that frequent changes are not beneficial. From the foregoing analysis I think you will agree that they could not be avoided. I think we are fully alive to our responsibility in the matter, and endeavour at all times to be fair and just to our officers, remembering their difficulties. Hoping you will give this the same publicity as your incorrect criticism, which would be quite welcome if deserved.

[We are glad that some of the changes have been due to the substitution of skilled for unskilled nurses. But for the rest our correspondent confirms the accuracy of our note.—Ed. The Hospital.]

### OUTDOOR UNIFORM.

"Sarah Gamp" writes: May I point out to the nurses who are at present concerning themselves about outdoor uniform that a good many babies' nurses at the present day are fully trained nurses, and therefore are quite entitled to wear hospital uniform, whether they wheel a perambulator or not; also that many fully-trained monthly nurses take their babies out in perambulators. In these days when monthly nurses are more often engaged for six weeks than a month, our babies are sometimes too heavy to carry far at the end of the month, and though fully-trained we prefer to wheel a perambulator rather than get over-tired. Also I often come across fully-trained nurses as babies' nurses in private families. A St. Thomas's nurse applied to take one of my babies "from the mouth" only a short time ago, and as she was to have no under she would have to wheel her baby about herself. And I think that the nurses who write about uniform are quite mistaken in thinking that we are taken so much notice of by the public when we go our walks. I wonder if more than one person in a hundred that we pass ever gives us a second thought and gives a thought as to whether we are fully trained or not. I fully agree with "Progress" and think that she has solved the problem better than anybody.

"An Experienced Nurse" writes: With respect to the uniform of hospital trained nurses, or those receiving training as nurses, being worn by nursemaids, I cannot understand why the hospital committees and matrons do not take the matter up. Why do they not all decide to sign a petition and send the same up to the Houses of Parliament to try if possible to get a Bill passed for prohibiting anyone else who is not a trained nurse or being trained as a nurse from wearing the same? I suppose that the wearing of uniform was first decided by the hospital committees and matrons, then why should they not try to protect the same, as no one else would be able to do it so effectually or cause it to be done? It certainly is appalling to see the number of nursemaids attired in the uniform of a trained nurse. Personally I wish the wearing of outdoor uniform was abolished. It is not a comfortable or convenient dress. The bonnet is no protection to the face and the circular cloak is most inconvenient to the private nurse when assisting a patient or walking with her in outdoor uniform. How much better would a neat tailor-made coat and skirt look with a plain hat to protect the face a little, than the circular, cumbersome cloak of to-day and the bonnet which is an apology for a head covering? In many institutions a material dress is worn when going out for off duty, and it would take no more time to put on a plain coat and skirt than to change the print gown for one of material. While the Bill for the registration of nurses is being considered this could be done at the same time, and last, but not least, all nursing homes and private nursing institutions should be put under Government inspection and supervision.

"Fever Trained" writes: The question of outdoor uniform being worn by the wrong people is continually being discussed, but no good results seem to come of these discussions. As there seems to be some reasons against State registration, could not a society be formed to supply badges to *bona fide* nurses which could only be obtained from the society through hospitals? I cannot imagine any hospital in the British Isles that would not join, and so make their staff members, and in that way prevent an outsider wearing the badge. The badges could bear the initial letters of the society's name and of the hospital to which the nurse belongs. Private nurses not attached to any hospital or institution could wear the badge of their training school, with the letters P.M.N. or P.G.N., as the nurse may be. Fever badges could be of a different colour. Could not some kind friend call a meeting of the heads of the various hospitals to organise a society? I feel sure that nurses would willingly subscribe to start the affair. Also could not the badges be registered, so that legal proceedings could be taken against anyone other than the authorised makers making and selling them, or against the authorised makers selling them to other people than the society? The hospitals invited to join would be the present recognised schools of general training and fever hospitals of a certain number of beds. Periodical examination by the society could also be held to enable nurses of the smaller hospitals, by paying a fee, to enter for the badge and being recognised as competent trained nurses. The badges could be slightly different for these nurses. Nurses trained in a recognised school, and not working in such at the present time, could wear the badge of their training school with some little distinctive mark. Plentiful newspaper notice would inform the public against employing other than badged nurses. In a few years the outdoor uniform could be all the same colour, stuff, and pattern, but a sudden change would mean a lot of waste.

### Presentations.

West Ham Infirmary.—A very pleasant evening was spent by the nurses and staff of West Ham Infirmary on Thursday last week, the occasion being the presentation to Dr. Blake, senior assistant medical officer at the Infirmary, on his marriage and removal to Walthamstow, of a silver tea and coffee service with waiter suitably inscribed, by the nursing staff and clerks; a case of silver spoons by the domestic staff; and a combination case of pickle fork, jam spoon, and butter knife by the head and assistant laundresses. Dr. Vallance, medical superintendent, made the presentation, and said that it was with pleasure that he presented the gifts to Dr. Blake on behalf of the staff. He regretted very much the loss of so valuable an assistant. Miss Kingston, to whom he was to be married, had been a nurse at West Ham Infirmary, and he could say that she was a very capable nurse, and always did her duty in a highly satisfactory manner. Dr. Blake was a fortunate man to have her for his wife. He gladly acknowledged the excellent way in which Dr. Blake had assisted him, and thanked the staff for giving him the privilege of presenting the gifts on their behalf. Dr. Blake, in reply, said that on behalf of Miss Kingston and himself he could not thank them enough for their kind expressions and gifts. He had only done his duty, and hoped that he should be as successful in his new life as he had been at the Infirmary. Miss Kingston joined him in all good wishes.

### Novelties for Nurses.

(By Our Shopping Correspondent.)

### ORLWOOLA.

Orlwoola is a beautiful woollen fabric which can be obtained at all drapers and is manufactured by the Leigh Mills Company, Stanningley. Many advantages can be claimed for the material. It is light and fine, extremely soft to the touch, it has a large and varied range of colour and design, and it is unshrinkable. For blouses, children's dresses, tennis shirts, and underwear it is most admirable.

### Appointments.

[No charge is made for announcements under this head, and we are always glad to receive and publish appointments. The information, to insure accuracy, should be sent from the nurses themselves, and we cannot undertake to correct official announcements which may happen to be inaccurate. It is essential that in all cases the school of training should be given.]

Bolingbroke Hospital, Wandsworth Common, London.—Miss Constance Mary Starling has been appointed sister. She was trained at St. Thomas's Hospital, London.

Northampton General Hospital.—Miss Annie M. Spaul has been appointed sister. She was trained at Norfolk and Norwich Hospital, and in midwifery at the Louise Margaret Hospital, Aldershot. She has also been on the private staff of the Norfolk and Norwich Hospital.

Princess Alice Memorial Hospital, Eastbourne.—Miss May Etchell has been appointed night sister. She was trained at Mill Road Infirmary, Liverpool. She has since been sister in charge at the same institution, and sister at the Highfield Infirmary, Knotty Ash, Liverpool. She has also done private nursing.

Queen Victoria Royal Infirmary, Preston.—Miss Frances C. Lorrimer has been appointed assistant matron. She was trained at Leeds General Infirmary and has since been charge nurse at Scarborough Hospital and Dispensary, and ward sister and night superintendent at the Royal Infirmary, Sheffield.

Rous Memorial Hospital, Newmarket.—Miss A. M. Tubby has been appointed matron. She was trained at the London Hospital, and has since been matron of Trowbridge Cottage Hospital.

Royal Cornwall Infirmary, Truro.—Miss Katharine Thorburn has been appointed sister. She was trained at the Derbyshire Royal Infirmary, Derby, and has since been sister at the Royal Victoria Hospital, Bournemouth. She holds the certificate of the Central Midwives Board.

Royal Infirmary, Dumfries.—Miss Amy Sharp has been appointed sister. She was trained at the Royal Albert Edward Infirmary, Wigan, where she has since been sister. She has also been sister at the Chelsea Hospital for Women, London, and temporary matron at Bradstock Lockett House, Southport.

St. Luke's Home for the Dying, Bayswater, London.—Miss B. Brooke Alder has been appointed matron. She was trained at the Children's Hospital, Great Ormond Street, London, and the Norfolk and Norwich Hospital. She served in South Africa during the war, and was afterwards sister at Sussex County Hospital, and Charing Cross Hospital, and matron of the Maitland Cottage Sanatorium, Peppard Common, Oxon.

Shoreditch Poor-law Infirmary, Hoxton Street.—Miss C. L. Elliott has been appointed home sister. She was trained at Birmingham Poor-law Infirmary. She has since been sister at the same institution, sister at Kingston-on-Thames Poor-law Infirmary, and night superintendent at St. Pancras Poor-law Infirmary. She holds the certificate of the Central Midwives Board.

Walton Workhouse Hospital.—Miss Fanny P. Spann has been appointed head night charge nurse. She was trained at Birkenhead Union Infirmary and has since been sister at the same institution. She holds the certificates of the Central Midwives Board and the Incorporated Society of Trained Maseuses.

Western Hospital, Fulham Road, London.—Miss Charlotte Alice Phipps has been appointed charge nurse. She was trained at Bristol Royal Infirmary, and was afterwards on the private staff. She has since been nurse at East Preston Infirmary, Sussex. She holds the certificate of the Central Midwives Board.

### A Book and its Story.

MEMOIR OF KATHERINE G. LOCH, R.R.C.*

Nearly twenty years ago, in March 1888, a little band of nursing sisters arrived in Bombay in charge of Miss Loch and Miss Osley, lady superintendents. This little company was the pioneer of the Indian Nursing Service instituted by Lady Roberts, with the consent of the Indian Government, for skilled nursing of sick soldiers. Lady Roberts recognised the need of some addition to the nursing staff of the military hospitals, which consisted until then of men only. In the preface, written by Lord Roberts, we read: "Miss Loch brought to her task enthusiasm and indomitable energy, and she was ably helped by a number of devoted lady nurses. The fruits of their work are to be seen in the military hospitals of India to-day." Queen Alexandra's Military Service for India is the outcome, as is generally known, of the movement due to Lady Roberts's exertions at the outset.

Miss Loch's memoir is compiled from letters written regularly every week to her sisters, which are full of interest. They give a vivid picture of her hospital life in India, impressions of places and things seen, and the long journeys taken, under great difficulties at times, when on administrative duty connected with her appointment. The excitements and anxieties of her official position required the exercise of infinite patience and tact. "By her administrative ability, strikingly sound and tactful common sense, and by her decisive and level-headed judgment in complex and trying circumstances, she had obtained the high esteem of the medical authorities with whom she was brought into communication. Undoubtedly under a less able and less judicious pioneer the Indian Nursing Service could not have achieved the marked success which justified the Government of India in adding to the personnel and extending the areas of usefulness." In addition to her ability to combat the friction and difficulties incident to the position, in connection with the military authorities and the nursing movement, she possessed the faculty of personally inspiring devotion in the sisters associated with her: "They were certain of being able to count upon just and impartial treatment when occasions arose that necessitated action being taken."

Some details of Miss Loch's training and earlier life may be interesting to readers of The Hospital: "She was the daughter of George Loch, Esq., Q.C., and of Catherine Brandreth, and was born on September 21, 1854, at Worsley Old Hall, Manchester." From the age of eighteen she had wished to become a nurse. But, as nursing at that date was not as it is now regarded—a vocation which women of all classes can enter without losing social position—her parents would not allow her to take it up until she was twenty-five. At this time she showed little of the mental power which was so conspicuous later; but tenacity of purpose was always there. She never wavered in her wish to become a nurse. She studied nursing theoretically in these waiting years from books, and on her twenty-fifth birthday she claimed the right to put into action the wish that had been dominant for so long. Her first acquaintance with hospital life was made in the Royal County Hospital of Winchester in 1879, when she became a probationer in the women's surgical ward. Mrs. A. Bolton, the sister then in charge of the ward, writes of Miss Loch: "Anyone who has had experience of what raw probationers are will understand how great is the relief of receiving one so bright and capable as she was. . . . Her training was before the time of the three years' certificate and probationers were taken for one year only. But that year was one of probation, and with hard work, bad food, and very little time off, was a pretty severe test of anyone's powers of endurance and enthusiasm in the work. . . . She was always bright and cheerful and much beloved by her patients and quite the life of the probationers' room. Everything that happened, whether pleasant or unpleasant connected with her work, was always received with interest as an experience. . . . I always have a feeling of pride that she began her training under me and think with satisfaction of the splendid work she eventually did."

In 1882 Miss Loch became night superintendent at St. Bartholomew's. Later she was placed in charge of a men's surgical ward, a post which she retained until the end of 1887. Among the members of the nursing staff, Miss R. A. Betty was one who became an intimate and devoted friend. The friendship between the two was broken only by death. Of the years between 1882 and 1887 Miss Betty writes: "Among her patients she laboured for them and made it one of the happiest and brightest wards. . . . She was always cheerful, even when things went wrong, but never depressed or cast down unless there was no hope." Night duty, which is so often tedious and trying to many, was to her specially interesting, for it is then that a nurse has special calls on those claims for prompt action and decisiveness which were characteristic of herself. "It seemed as though in the still, silent watches, when the patients are at their worst . . . that she felt the reality of what she could be to those who were helpless and dependent on her for the relief and comfort she could and would give them. . . . In these days a three or four years' training is made a *sine qua non*, and no doubt is most essential; but I cannot help noticing that she with her one year's training at Winchester Hospital was capable of so much more than so many I have met after their long term of training and experience." During 1887 it was announced that the India Office had decided to send out nursing sisters to work in the military hospitals in India. Miss Loch was at once anxious to join the scheme. To go to India had been the wish of her life, and "To go as the pioneer of a great and good work stimulated all her vigour and courage to be part of it, whatever might happen." Miss Loch's comments on the movement are characteristic of the brave optimism which inspired her conduct.

"It must and it will succeed; nothing which is so much wanted can fail."

We regret that space will not permit us to enter on Miss Loch's work in India. Our readers, we hope, will be able to follow it in her memoir for themselves. It cannot fail to be both helpful and absorbing to them. A sick officer's tribute to lady nurses on duty in the Indian military hospitals must close this notice: "The continual presence of these ladies has raised the tone and morale of the staff, and the check which soldiers now have to exercise on their conduct during their temporary stay in hospital does, I am sure, benefit them for the remainder of their lives. Thanks to these lady nurses many a last message reaches the aged mother or the wife, sweetheart, brother or sister in England that otherwise would never have been uttered, but for the presence of a woman by a dying soldier's bedside. It must also be a relief to those at home to know that their son's last moments in a distant land are brightened by the tender influence and Christian sympathy of good women, and that they go to their long rest better, more contrite, and happier from the contact."

Miss Loch died in England at the comparatively early age of forty-nine. Her illness was primarily caused by the excessive mental strain acting upon a constitution weakened by long residence in India.

* Edited by Surgeon-Major-General Bradshaw, M.A., C.B. (London: H. Frowde. 6s.)

### TRAVEL NOTES AND QUERIES.

By our Travel Correspondent.

A Fortnight in Belgium (Beetle).—This information was published on March 31.

Information Several Years Old (Wanderer).—No; you must not rely upon what I told you five years ago. Prices have risen and hotels changed hands. (1) You can do it on the sum you have. (2) The General Steam Navigation Company has good boats and well appointed. You must go by that route if you intend to keep within the amount you mention. (3) Bruges is a much better centre for your purpose than Knokke. When you have decided your tour I will give you addresses.

Edinburgh for a Week (Mina).—Your project is, unfortunately, out of the question. The journey alone would take all the money. I think you would like what I am about to propose. Go to the Bay of St. Malo to Mrs. Dyott, Villa Olga, St. Malo, France. You would go by boat from Southampton, so that would be conveniently near for you. Second-class return £1 5s. Mrs. Dyott's house is near to the Quay, so that expense at that end would be small. On the money you have for your excursion you could stay a fortnight at St. Malo. Write to Mrs. Dyott (using my name) on a prepaid postal letter card; ask her terms and if she can receive you at the date you need. Write early, as her house is very full.

Leipzig and Dresden (Arrange).—We must hope to be as successful as in 1905. There is no cheaper way than by the Hook. Second-class return to Dresden £4 14s. 4d.; but why not go third class, it will make probably 30s. or 35s. difference? Why stay at Leipzig? Meet your daughter there and take the next train to Dresden. If a halt is compulsory at Leipzig go to Hôtel Hentschel—terms four or five marks per day—or to Hoffman's Hôtel Garni, 14 Wintergarten Strasse, where you can have cheap rooms and take your meals out. At Dresden go to the Münchner Hof, 21 Kreuz-Strasse, or the Amalien Hof, 24 Amalien, Strasse, both very cheap. Always ask for rooms on the third or fourth floor. Tell me if I can help further.

A Fortnight on the Rhine (Ignoramus).—You have enough money to do it very comfortably. Return ticket to Heidelberg, second class, is £3 15s. 6d.; it will enable you to go up the Rhine by steamer from Cologne to Mainz and to return by rail, which saves time. Route, *via* Harwich and the Hook. Spend one night at Cologne.—Hôtel Rheinischer Hof unter Fetterhennen, close to the Cathedral. Take the steamer the next morning (breakfast on board) to Konigswinter. Go to Hôtel Restaurant Mattern; stay there three nights and visit the seven mountains. Then steamer to Coblentz; stay three nights at Hôtel de Trêves in the Clemens Platz, or, if full, at Hôtel Springer. Many excursions to be made here. Then steamer again to Mainz. By taking the morning boat you need not remain a night at Mainz, but go straight on to Heidelberg; put up at Pension Anglaise, 49 Anlage, or, if full, at Pension Internationale, 22 Anlage. Stay five nights. This makes your tour twelve days, not counting the two spent on the sea. One day will be occupied in the rail from Heidelberg to the Hook. I have reckoned your expenses at 6 marks a day (Heidelberg not more than five); this leaves you £2 2s. 6d. for excursions and tips. If you think this is running it too closely, stay one day less at Coblentz. Always ask for rooms on the third or fourth floor.

Rules in Regard to Correspondence for this Section.—All questioners must use a pseudonym for publication, but the communication must also bear the writer's own name and address as well, which will be regarded as confidential. All such communications to be addressed "Travel Correspondent, 28 Southampton Street, Strand." No charge will be made for inserting and answering questions in the inquiry column, and all will be answered in rotation as space permits. If an answer by letter is required, a stamped and addressed envelope must be enclosed, together with 2s. 6d., which fee will be devoted to the objects of "The Hospital" Convalescent Fund. Ten days must be allowed before an answer can be published.

### Notes and Queries.

### REGULATIONS.

**The Editor is always willing to answer in this column, without any fee, all reasonable questions, as soon as possible.**

**But the following rules must be carefully observed.**

**1. Every communication must be accompanied by the name and address of the writer.**

**2. The question must always bear upon nursing, directly or indirectly.**

**If an answer is required by letter a fee of half-a-crown must be enclosed with the note containing the inquiry.**

*School Inspectorship.*

(58) Can you tell me how to obtain a post on the London County Council as nurse-inspector of school children? To whom should I write? And is it necessary for a fully-trained nurse to train further?—*B. M.*

These posts are not easy to obtain. Write for particulars to the Clerk to the London County Council, Spring Gardens, S.W.

*Tubercular Spine.*

(59) Where can patient suffering from tubercular spine be received for less than £1 per week?—*Anxious.*

Write to Secretary, Richmond House, Clare, Suffolk. If unsuccessful, consult list of sanatoria in "Medical Homes," Scientific Press, 28 Southampton Street, Strand, price 7d. post free.

*Hysteria.*

(60) Can you tell me where lady suffering from hysteria needing observation could be received?—*Acton.*

We fear that the only mental home where patient would be received at so low remuneration would be in private department of some of the County Asylums. The cost is usually from 14s. to £1 1s. There is a list in Burdett's "Hospitals and Charities," The Scientific Press, 28 Southampton Street, Strand.

*Training.*

(61) Are there any children's hospitals where young girls are trained as nursemaids?—*Children.*

The Norland Institute, 10 Pembridge Square, W., send young girls whom they train to a children's ward in a hospital for a time.

*Bournemouth.*

(62) Can you assist me to secure a letter for Convalescent Home in Bournemouth?—*S.*

You will not require a letter if you can secure admission at St. Joseph's Convalescent Home, Branksome Wood Road, where the cost is 8s. per week. Write to the Lady Superintendent.

*Certificate.*

(63) Is it any use answering an advertisement for trained nurse, as I have had training but no certificate?—*M.*

In some cases you might be engaged, especially if you have a satisfactory testimonial from your Matron. It is a disadvantage to train at an institution which does not give a nursing certificate.

*Convalescent Home for Nurses.*

(64) Can you tell me of a convalescent home where my sister, who is a nurse, would be received free, after illness?—*F. D.*

This journal has a convalescent fund, maintained by its readers, to help such cases as your sister's. Write to the Secretary, "Hospital Convalescent Fund," 28 Southampton Street, Strand, W.C.

*Governess.*

(65) Where can I train as a nurse without payment of a large premium?—*E. M. C.*

Get "How to Become a Nurse," The Scientific Press, 28 Southampton Street, Strand. Therein you will find all the information you require.

*The Norland Institute.*

(66) Please give me the address of the Norland Institute for Nurses.—*L. J.*

The address is 10 Pembridge Square, London, W.

*Training as Masseur.*

(67) Can you tell me of an institute in which a man could be trained as a masseur, or could he get private lessons in massage?—*Guy's Trained Nurse.*

He could receive instruction in massage at the National Hospital for the Paralysed and Epileptic, Queen Square, Bloomsbury. The Secretary, The Incorporated Society of Trained Masseuses and Masseurs, 12 Buckingham Street, Strand, W.C., would probably advise about private lessons.

*South Australia and Cairo.*

(68) Can you give me names of nursing homes in South Australia and Cairo?—*Anxious No. 2.*

The Nurses' Home, North Terrace, Adelaide. For Cairo the Matron of the Station Hospital may help you.

*The Medico-Psychological Society.*

(69) How can I obtain the medal of the Medico-Psychological Society, as my asylum authorities will not grant it to me, though I have the certificate?—*A Male Nurse.*

Write to the Society, state your case, and possibly they may allow you to buy the medal yourself.

*An Awkward Position.*

(70) I belong to an association which trained me free, and now I find I am not strong enough to do the work. If I am ill again and refuse to continue, can they keep my Central Midwives Board certificate unless I repay for the training?—*Cottage Nurse.*

The position is awkward, but we think it largely depends upon yourself to solve the difficulty. If you are tactful, and get your doctor to explain that you cannot do the work, even though you may wish to, it is improbable that the association will act unkindly. It will be a pity if you cannot continue such useful work.

*Excision of the Spleen.*

(71) How long may a patient live after excision of the spleen? and is it only a very rare operation?—*Inquisitive.*

We do not answer medical questions of this description.

*Deafness.*

(72) Is it possible for a person who is slightly deaf to become a hospital nurse?—*Puzzled.*

Even if you might be accepted in a hospital, it would be a grave mistake to take up nursing as a profession. A feeble patient would be sorely tried if a nurse could not hear well.

*General Hospital.*

(73) I have lost my post owing to illness. I am only 21, and would like to be in large general hospital.—*Only a Pro.*

You had better wait until you are old enough—23—to enter a general hospital. Any training you get before then is a disadvantage, as most matrons prefer those without previous training. We are sorry we have not space for your poetry.

*Home of Rest.*

(74) Can you tell me of a home of rest for nurses free?—*Rest.*

There is no free home of rest for nurses during the summer months, but if you write to the Secretary of the "Hospital" Convalescent Fund, 28 Southampton Street, Strand, she may be able to help you.

*Canada.*

(75) Where can I apply to obtain appointment as nurse in Canada?—*M.*

You do not say whether you wish for hospital or private nursing. Write Matron, General Hospital, Montreal; Matron, General Hospital, Winnipeg, Manitoba; or Victorian Order of Nurses for Canada, Ontario.

*Post in United States.*

(76) How can I obtain a post in the United States?—*Manchester.*

You will find it very difficult to do so. You might write to the Matron of one of the large hospitals, such as the Presbyterian Hospital, Maddison Avenue, New York.

*Fever Hospital Probationer.*

(77) (1) What is the earliest age for a probationer in a fever hospital? (2) Is it the duty of the matron to supervise the disinfection of letters? (3) Can a matron prevent a probationer writing hospital news to her friends?—*F. H. S.*

(1) Twenty-two or twenty-three. (2) It is the duty of some official to see that letters are disinfected, but not necessarily the matron. (3) A matron would not forbid, but she might ask and advise probationers not to gossip about the hospital.

*Nursing Homes Abroad.*

(78) Will you give me addresses of nursing homes in France, Italy, or Switzerland?—*X. Y. Z.*

English Nurses' Home, Villa St. Joseph, Biarritz; Nice Nursing Institute, Villa Pilatte, Avenue Desombrois, Nice, France; Institute for Trained English Nurses, Sunny Bank, San Remo, Italy.

**Handbooks for Nurses.**

| | Post Free. |
|---|---|
| "How to Become a Nurse: How and Where to Train." | 2s. 4d. |
| "Nursing: its Theory and Practice." (Lewis.) ... | 3s. 6d. |
| "Nurses' Pronouncing Dictionary of Medical Terms." | 2s. 6d. |
| "Complete Handbook of Midwifery." (Watson.) ... | 6s. 4d. |
| "Preparation for Operation in Private Houses." ... | 0s. 6d. |

**Of all booksellers or of The Scientific Press, Limited, 28 & 29 Southampton Street, Strand, London, W.C.**

### For Reading to the Sick.

"LEAD ME."

Lead me, Almighty Father, Spirit, Son,
Whither Thou wilt, I follow, no delay.
My will is Thine, and even had I none,
Grudging obedience, still I would obey.
Faint-hearted, fearful, doubtful if I be,
Gladly or sadly I will follow Thee.

Into the land of righteousness I go,
The footsteps thither Thine, and not my own,
Jesu, Thyself the way, alone I know
Thy Will be mine, for other have I none.
Unprofitable servant though I be,
Gladly or sadly let me follow Thee.

*Bishop Stubbs.*

Depression often springs from the fact that, seeking God, we do not find sufficient consolation in the search. The desire to feel His comfort is not the same as the desire to possess Him; for the former may spring wholly from self-love. Our prostrate and disheartened nature is not content with faith only, and wants to see its own progress. Our pride is disgusted at our faults, and we mistake this disgust for true repentance. Our self-love would fain have the gratification of seeing itself perfect: we are vexed with ourselves, and are impatient and out of temper both with ourselves and others. Grievous error! As though our vexation would forward God's work, or as though we should approach nearer to the God of peace by destroying our inward peace! Martha, Martha! why art thou troubled about so many things in the service of Jesus Christ! But one thing is needful; to love Him, and remain patiently at His feet.—*Fénélon.*

It is His part to be specially "The Comforter"; to enter into the heart, which, knowing its own bitterness, is brooding apart over its sorrows, or quailing at the thought that it is called upon to bear sufferings, anxieties, and cares, which others do not, and cannot enter into; and to teach that heart that it has not been left by God to suffer alone. For lo! God Himself is with it, filling its darkest and most desolate chamber with a presence, the brightness of whose glory and the power of whose might, not even the pillar of the cloud, or the cloven tongues like as of fire, can adequately represent; and showing even to the most friendless of this world that they have a truer source of consolation, always with them, than all the sympathy of this world, even the most genuine, the most loving, can afford.—*A. P. P. C.*

What a difference it makes to us; as the bright sunshine floods the landscape, and colour mounts up in the flowers, and the blue sky is radiant in unclouded beauty; the hard path seems easier, the very raindrops sparkle in the light, and the distance opens out before us, like Hope itself, bidding us look up and look onwards, and to raise our eyes from the slippery path and the frequent faltering of our weary feet, to the road before us which leads onward and leads home.—*Canon Newbolt.*

